# Supplementary material for: Seminoma and Embryonal Carcinoma Footprints Identified by Analysis of Integrated Genome-Wide Epigenetic and Expression Profiles of Germ Cell Cancer Cell Lines
Source: PLoS One. 2014 Jun 2;9(6):e98330. doi: 10.1371/journal.pone.0098330 (PMC4041891; doi:10.1371/journal.pone.0098330)
Supplement: File S1 — ZIP file containing DMRforPairs output for significant regions. Please start from the html files. (ZIP) [file pone.0098330.s008.zip › figures/12280.pdf]

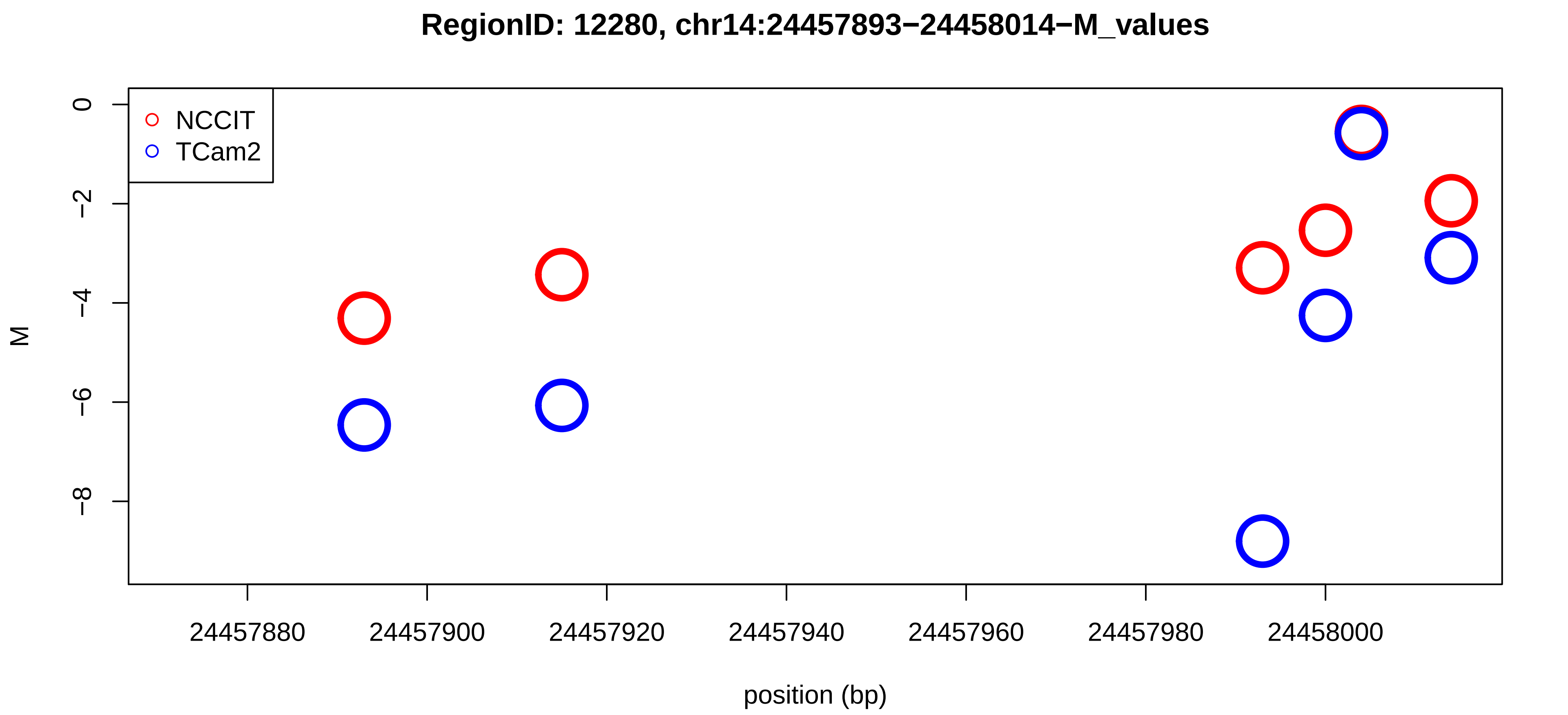
RegionID: 12280, chr14:24457893−24458014−M_values
NCCIT
TCam2
M
0
−2
−4
−6
−8
24457880
24457900
24457920
24457940
24457960
24457980
24458000
position (bp)

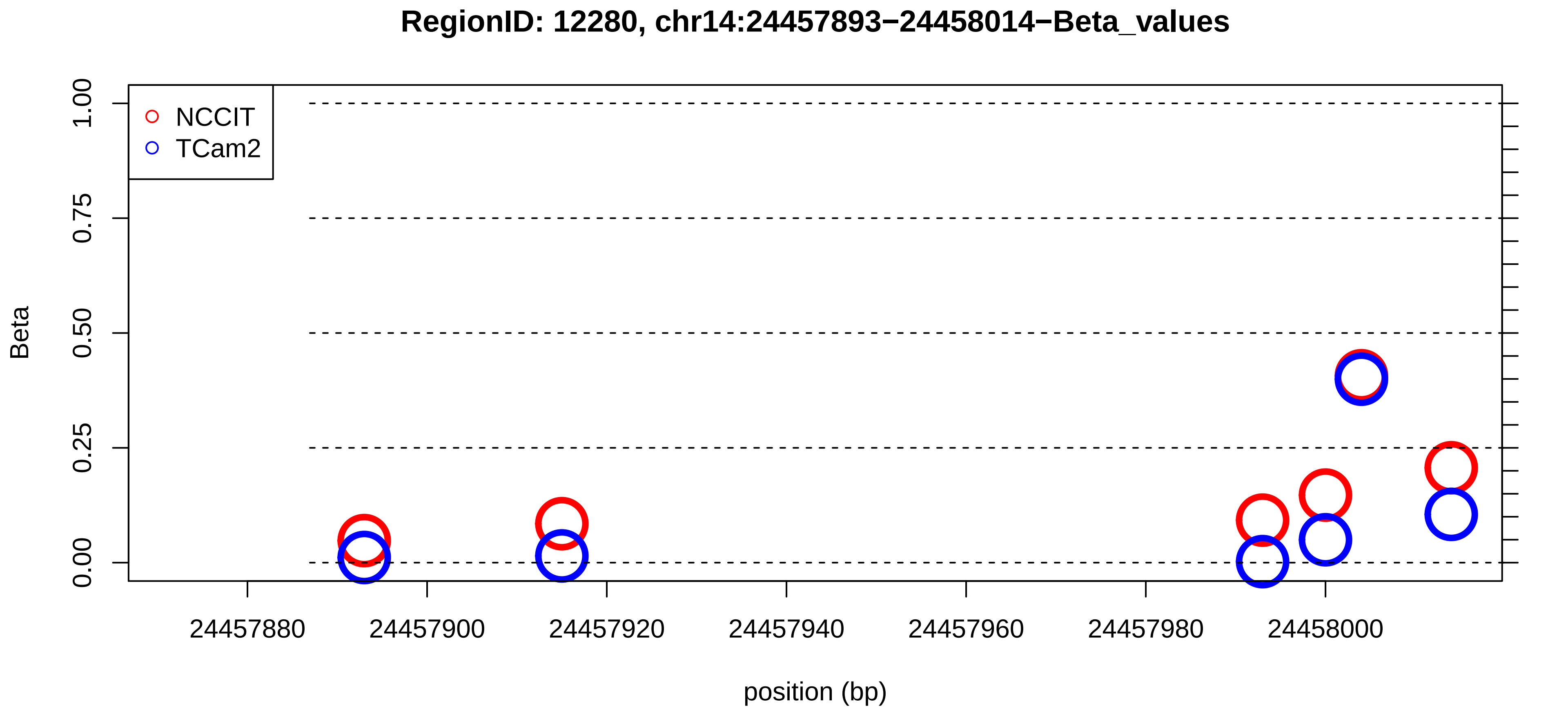
RegionID: 12280, chr14:24457893−24458014−Beta_values
NCCIT
TCam2
Beta
1.00
0.75
0.50
0.25
0.00
24457880
24457900
24457920
24457940
24457960
24457980
24458000
position (bp)
